# Xeroform Gauze Bolster: A Cost-Effective Alternative to Synthetic Skin Grafts

**DOI:** 10.7759/cureus.45307

**Published:** 2023-09-15

**Authors:** Kelsey L Johnson, Nicole Papac, Jason M Hirshburg

**Affiliations:** 1 Dermatology, Oklahoma University Health Sciences Center, Oklahoma City, USA

**Keywords:** skin graft alternative, cost-effective, mohs syrgery, grafting, synthetic skin graft, xeroform, bolster

## Abstract

This case presentation describes and justifies the use of petrolatum gauze that is impregnated with 3% bismuth tribromophenate (Xeroform) as a low-cost and effective alternative for synthetic skin grafts. Herein, we discuss clinical trials that demonstrate the benefits of utilizing Xeroform for second-intention healing as well as photographs of a case in which the authors used this method and followed a patient's wound-healing process over time.

## Introduction

Synthetic skin grafts such as porcine xenografts have been utilized for Mohs surgery defect repairs for many years as they are thought to decrease healing time and infection rates as compared to second-intent healing alone; however, they can be cost-prohibitive [1]. Petrolatum gauze that is impregnated with 3% bismuth tribromophenate (Xeroform) is cheap and may be helpful as a possible alternative with similar benefits for second intention healing of surgical defects. A head-to-head clinic trial comparing a porcine xenograft to second-intent healing was registered on ClinicalTrials.gov in 2019 but was ultimately terminated as the company manufacturing the xenograft discontinued production [2]. Numerous brands of porcine xenografts have been removed from the market in recent years, and the remaining synthetic skin grafting materials available have a high cost for the healthcare system and most patients [3]. Fortunately, Xeroform gauze may be an inexpensive and widely available solution to this problem.

## Case presentation

**Figure 1:**
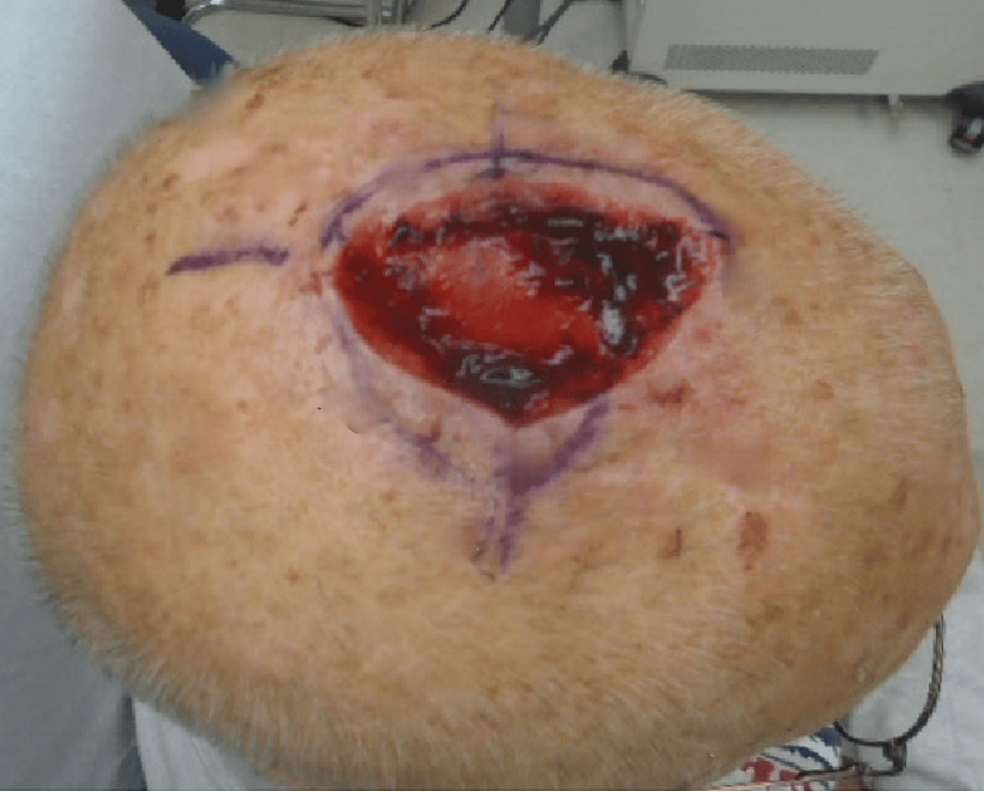
Final Mohs surgical defect on the vertex scalp

**Figure 2:**
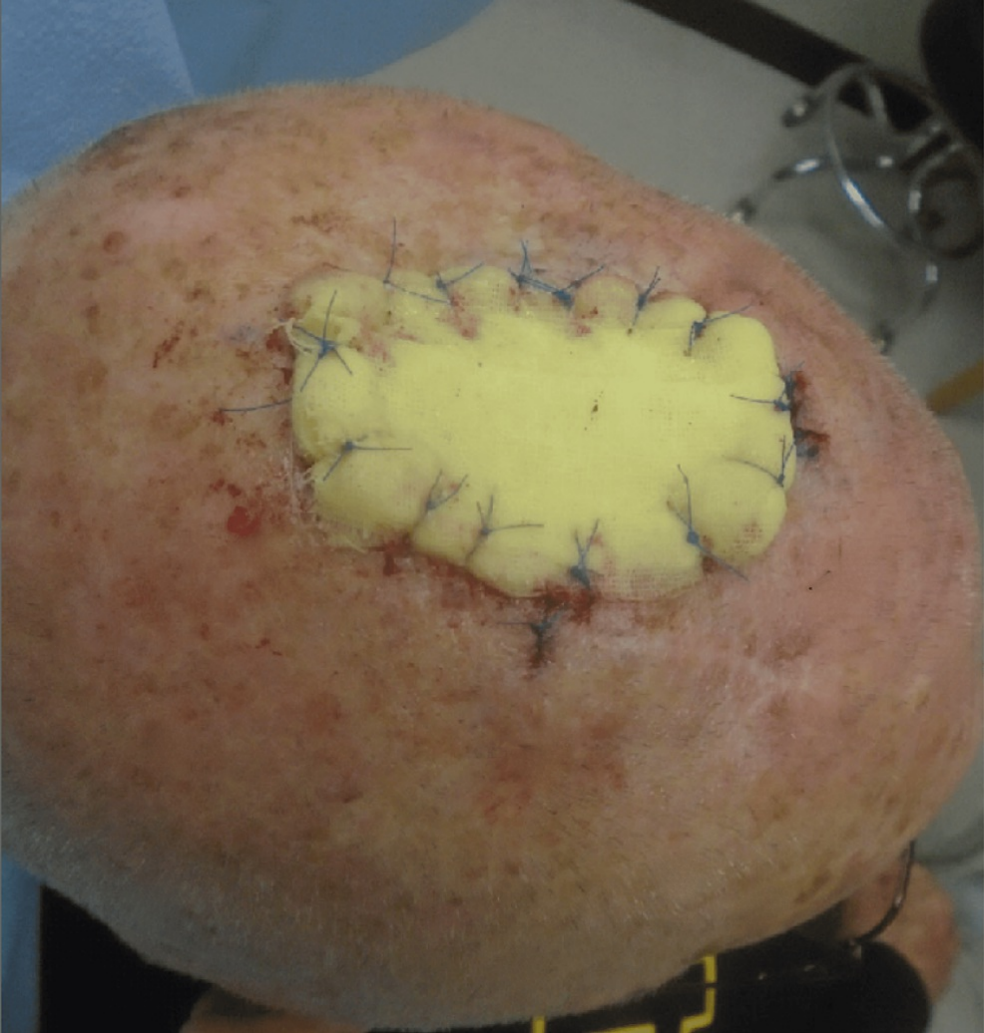
Xeroform gauze bolster sewn into place

**Figure 3:**
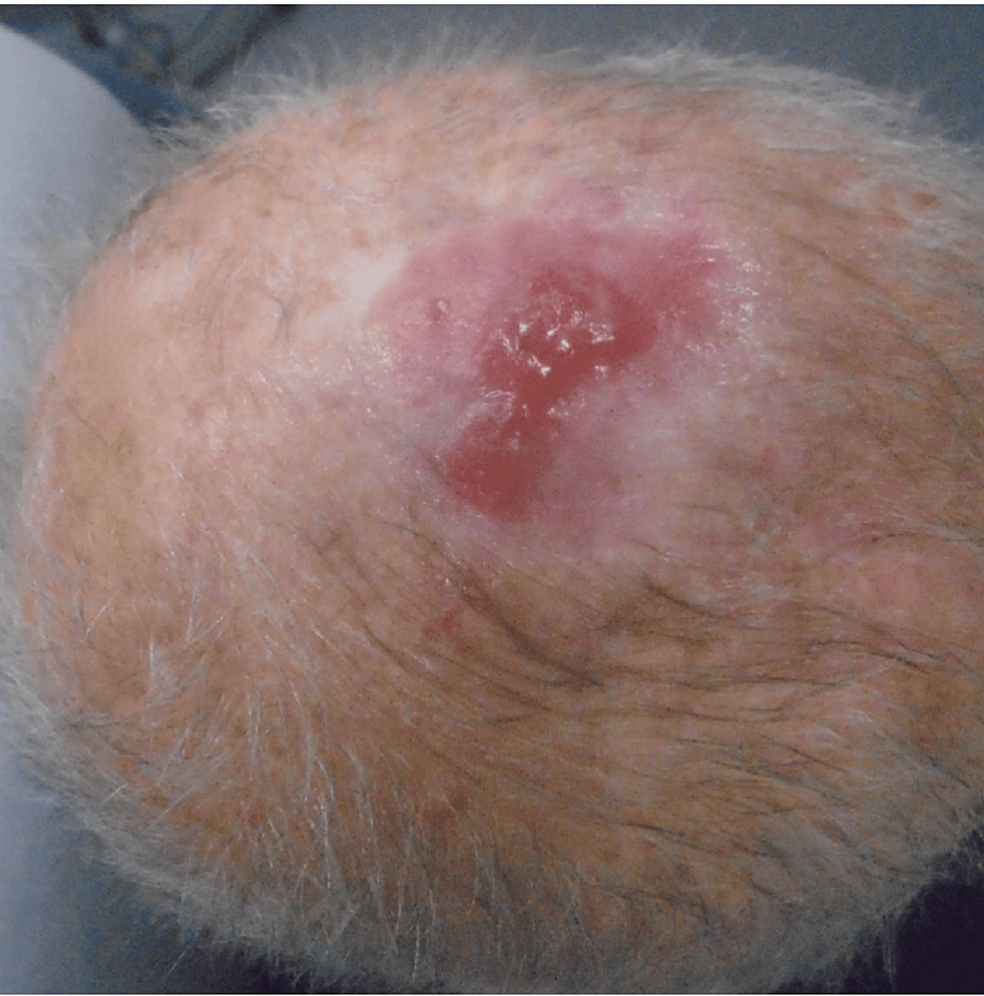
Healthy granulation tissue at one-month wound check

An 85-year-old Caucasian male with a personal history of non-melanoma skin cancer in the setting of chronically sun-damaged skin presented to the clinic for surgical evaluation and treatment of a biopsy-proven melanoma in situ involving the vertex scalp. After a discussion of treatment options, the decision was made to proceed with staged excisions with en face margin evaluation (“slow Mohs”). The tumor was cleared after two stages resulting in a 5.7 x 6.0 centimeter defect (Figure 1). The patient’s goals in repair were minimizing the risk of infection and required wound care. The decision was made to sew into place Xeroform gauze as a bolster (Figure 2). The bolster was removed after two weeks, and the patient followed up four weeks after surgery for a wound check where he was found to have healthy granulation tissue at the site of the wound (Figure 3).

## Discussion

We have found that suturing Xeroform gauze into place as a wound dressing can be helpful with wound healing in our Mohs patients. Xeroform has demonstrated rapid reepithelialization, while also being easy to use, resistant to infection, and the most cost-effective material when compared to Opsite (polyurethane film), Kaltostat (calcium sodium alginate), DuoDERM (hydrocolloid), Aquacel (hydrofiber), and Mepilex (silicone foam) [4]. In a study that devised a scoring system evaluating percent reepithelialization, inflammation, infection, ease of use, and cost for various materials, Xeroform tied for first rank with DuoDERM. Xeroform was found to be slightly inferior to DuoDerm in terms of reepithelialization rate [4,5]. Additionally, as in the case presented here, Xeroform can be sutured into place. Thus, in areas where slower rates of reepithelialization can be tolerated or there is a concern for the ability of the patient to care for the wound, Xeroform may be the preferred wound care material.

Xeroform is commonly used to wrap pedicle flaps in Mohs repairs [6]. Additionally, the use of tie-over bolsters with Xeroform has been described in the literature to promote second-intent healing [5]. As illustrated with this case report, the authors have found wound healing by second intention following Mohs surgery to be another potential beneficial application of Xeroform gauze. We removed the Xeroform bolster after one to two weeks which ensured the granulation tissue did not adhere to the mesh.

## Conclusions

Both effectivity and affordability should be considered when deciding what materials will be used for second-intention healing. Xeroform gauze bolsters in place of synthetic skin grafts have been noted to be an effective alternative to synthetic skin grafts. Dermatologic and Mohs surgeons should consider this simple and affordable repair technique before pursuing more cost-prohibitive options.
